# Genome Sequence of Pseudomonas chlororaphis Lzh-T5, a Plant Growth-Promoting Rhizobacterium with Antimicrobial Activity

**DOI:** 10.1128/genomeA.00328-18

**Published:** 2018-05-03

**Authors:** Zhenghua Li, Xiaoming Li, Qiangcheng Zeng, Mei Chen, Dan Liu, Jihua Wang, Liang Shen, Feng Song

**Affiliations:** a Shandong Provincial Key Laboratory of Biophysics, Institute of Biophysics, Dezhou University, Dezhou, China; b College of Physics and Electronic Information, Dezhou University, Dezhou, China; c College of Agronomy, Shandong Agricultural University, Tai’an, China; d College of Life Sciences, Dezhou University, Dezhou, China; e Dezhou Number One Middle School, Dezhou, China

## Abstract

Pseudomonas chlororaphis Lzh-T5 is a plant growth-promoting rhizobacterium (PGPR) with antimicrobial activity isolated from tomato rhizosphere in the city of Dezhou, Shandong Province, China. Here, the draft genome sequence of P. chlororaphis Lzh-T5 is reported, and several functional genes related to antifungal antibiotics and siderophore biosynthesis have been found in the genome.

## GENOME ANNOUNCEMENT

Rhizobacteria referred to as plant growth-promoting rhizobacteria (PGPRs) (1, 2), which have antimicrobial activity and could influence plant growth, have the potential for use as bioagents in controlling plant diseases and improving crop production. Pseudomonas chlororaphis is a widespread bacterium in the rhizosphere soil which can produce phenazine antibiotics that exhibit antifungal activity (3–6).

Strain P. chlororaphis Lzh-T5 was isolated from tomato rhizosphere in an area affected by root rot, a fungal disease caused by Fusarium moniliforme. Here, we report the draft genome sequence of P. chlororaphis Lzh-T5. Genomic DNA of P. chlororaphis Lzh-T5 was extracted and then sequenced using the PacBio and Illumina MiSeq systems, respectively.

The raw data were filtered and assembled by SPAdes software version 3.9.0 (7) and A5-MiSeq version 20150522 (8) to generate 1,224 Mb of total clean data, and the genome coverage was 164.0×. The assembled genome of P. chlororaphis Lzh-T5 comprises a single circular chromosome of 6,826,693 bp in length, with a GC content of 63.06%. Its genome comprises 6,282 genes, 6,116 open reading frames (ORFs), 67 tRNA genes, and 16 rRNA genes, which is similar to P. chlororaphis PA23 (99%) (GenBank accession number CP008696), P. chlororaphis ATCC 13985 (99%) (GenBank accession number LT629738), and P. chlororaphis LMG 21630 (98%) (GenBank accession number LT629747).

Like most Pseudomonas spp., P. chlororaphis Lzh-T5 possessed several gene clusters to generate siderophores, such as pyoverdine (PVD) and achromobactin. There were two nonadjacent clusters involved in PVD synthesis, including 4 nonribosomal peptide synthetase (NRPS) genes (GenBank accession numbers CXP47_RS20395, CXP47_RS20400, CXP47_RS20405, and CXP47_RS20875) (9). Clusters to synthesize achromobactin consisted of the synthesis genes *acsA-F* (CXP47_RS15760, CXP47_RS15765, CXP47_RS15770, CXP47_RS15780, CXP47_RS15785 and CXP47_RS15790) and achromobactin transporter genes *cbrA* through *cbrD* (CXP47_RS15755, CXP47_RS15750, CXP47_RS15745, and CXP47_RS15740) (10).

P. chlororaphis Lzh-T5 can also synthesize antifungal antibiotics, and gene clusters for antibiotics synthesis were found. For example, *prnA* through *prnD* (CXP47_RS17640, CXP47_RS17635, CXP47_RS17630, and CXP47_RS17625) coding for pyrrolnitrin (11), *hcnA* through *hcnC* (CXP47_RS12080, CXP47_RS12085, and CXP47_RS12090) coding for volatile compound hydrogen cyanide (12), and *phzI*, *phzR*, *phzA through phzG*, *and phzO* (CXP47_RS25500, CXP47_RS25505, CXP47_RS25510, CXP47_RS25515, CXP47_RS25520, CXP47_RS25525, CXP47_RS25530, CXP47_RS25535, CXP47_RS25540, and CXP47_RS25545) coding for phenazine, were found (3). Meanwhile, as nitrogen-containing pigments, phenazine and its derivatives also have many biotechnological applications, such as colorimetric redox indicators (13).

In conclusion, the genome sequence and annotation of P. chlororaphis Lzh-T5 contributed to revealing the molecular mechanism of its antimicrobial activity, which suggests that P. chlororaphis Lzh-T5 could be used as a biocontrol agent of various soilborne diseases.

### Accession number(s).

The whole-genome shotgun project of Pseudomonas chlororaphis Lzh-T5 has been deposited at GenBank under the accession number CP025309.
